# A convolutional neural network approach for IMRT dose distribution prediction in prostate cancer patients

**DOI:** 10.1093/jrr/rrz051

**Published:** 2019-07-19

**Authors:** Tomohiro Kajikawa, Noriyuki Kadoya, Kengo Ito, Yoshiki Takayama, Takahito Chiba, Seiji Tomori, Hikaru Nemoto, Suguru Dobashi, Ken Takeda, Keiichi Jingu

**Affiliations:** 1 Department of Radiation Oncology, Tohoku University Graduate School of Medicine, Sendai, Japan; 2 Department of Radiology, National Hospital Organization Sendai Medical Center, Sendai, Japan; 3 Department of Radiological Technology, School of Health Sciences, Faculty of medicine, Tohoku University, Sendai, Japan

**Keywords:** deep learning, convolutional neural network, radiation therapy, intensity-modulated radiation therapy, dose prediction, prostate cancer

## Abstract

The purpose of the study was to compare a 3D convolutional neural network (CNN) with the conventional machine learning method for predicting intensity-modulated radiation therapy (IMRT) dose distribution using only contours in prostate cancer. In this study, which included 95 IMRT-treated prostate cancer patients with available dose distributions and contours for planning target volume (PTVs) and organs at risk (OARs), a supervised-learning approach was used for training, where the dose for a voxel set in the dataset was defined as the label. The adaptive moment estimation algorithm was employed for optimizing a 3D U-net similar network. Eighty cases were used for the training and validation set in 5-fold cross-validation, and the remaining 15 cases were used as the test set. The predicted dose distributions were compared with the clinical dose distributions, and the model performance was evaluated by comparison with RapidPlan™. Dose–volume histogram (DVH) parameters were calculated for each contour as evaluation indexes. The mean absolute errors (MAE) with one standard deviation (1SD) between the clinical and CNN-predicted doses were 1.10% ± 0.64%, 2.50% ± 1.17%, 2.04% ± 1.40%, and 2.08% ± 1.99% for D_2_, D_98_ in PTV-1 and V_65_ in rectum and V_65_ in bladder, respectively, whereas the MAEs with 1SD between the clinical and the RapidPlan™-generated doses were 1.01% ± 0.66%, 2.15% ± 1.25%, 5.34% ± 2.13% and 3.04% ± 1.79%, respectively. Our CNN model could predict dose distributions that were superior or comparable with that generated by RapidPlan™, suggesting the potential of CNN in dose distribution prediction.

## INTRODUCTION

In recent decades, the quality of radiotherapy, such as intensity-modulated radiation therapy (IMRT) and volumetric-modulated arc therapy, has been greatly improved by inverse planning; these high-quality radiotherapy treatment techniques can prescribe a sufficiently high dose to the target while sparing normal tissues [1–5]. However, these planning techniques have several disadvantages. First, the plan optimization process is time-consuming [6, 7]: the planner optimizes each plan through a repeated trial-and-error process to meet the target and organs-at-risk (OAR) dose criteria. Second, in each patient, the achievable dose–volume histogram (DVH) is unknown at the time of optimization, and the dose constraints commonly use recommended values from previous studies, such as the quantitative analyses of normal tissue effects in the clinic (QUANTEC) guidelines [8], in which tolerance doses were defined by population data. Thus, plan quality and planning times determined by inverse planning depend on the skills and experience of planners and institutions [9–11].

RapidPlan^TM^ (Varian Medical Systems, Palo Alto, CA) is an artificial intelligence-based commercial software that was developed to improve plan consistency and planning efficiency; it is an atlas-based machine learning model in which a group of representative plans is used as a base model. The model is built by regression analysis, and the DVH objectives are derived; the model correlates the geometric and dosimetric relationships defined by human knowledge between the target and the OAR with the DVH of a clinical dataset. For each new patient, the model estimates the achievable OAR-DVH ranges, and the provided DVH objectives are used to perform the inverse planning optimization process. Several studies reported that the performance of RapidPlan^TM^ was comparable with that of manually optimized plans for different treatment techniques and that the sites and sub-potential manual plans could be improved with RapidPlan^TM^ [12–16]. However, some studies also reported that this algorithm could not be applied to automation for all treatment plans [17, 18] and that it was still limited by the inherent information present in the hand-crafted features. In addition, the feature quantity could only capture low-level features, and this algorithm was not sufficiently accurate for prediction [19].

To reduce dependence on hand-crafted features, we investigated a convolutional neural network (CNN) approach that specializes in image processing based on deep learning. Deep learning can automatically abstract and extract low-, mid-, and high-level features directly from the dataset to combine from end-to-end. Thus, by inheriting these functions, CNN can utilize spatial and structural information effectively for 2D or 3D image data with no human intervention [20]. In other words, CNN can automatically abstract and extract good features without a considerable amount of engineering skill or domain expertise, a key advantage of CNN. Because of this important feature, CNN may be able to make full use of imaging information.

Several studies demonstrated that the performance of CNN was comparable with those of the state-of-the-art methods and human performance for radiotherapy such as automatic segmentation [21–23], deformable registration [24], quality assurance [25], synthetic computed tomography (CT) generation [26, 27], response-to-treatment [28], and toxicity prediction [29] among others. However, there is not enough evidence that CNN can be used as a method to predict dose distribution [30]. Therefore, in this study, we evaluated CNN for its utility and efficacy as a method for predicting dose distribution and compared with the machine learning approach implemented in commercially available software [31–33]. Specifically, we evaluated the 3D CNN approach in prediction of IMRT dose distribution using only contours in the planning CT for prostate cancer by comparing with RapidPlan^TM^ [31–33], a conventional machine learning method.

## MATERIALS AND METHODS

### Patients and treatment planning

A total of 95 patients with prostate cancer who were treated with IMRT and selective urethral dose reduction between 2011 and 2018 were elected from the database of our institution. We studied all patients meeting the following inclusion criteria: the urethral catheter was inserted; the prescribed dose was 78 Gy or 80 Gy; there was all required data (e.g., contours’ data) for analysis. The prescriptions were 78 Gy in 39 fractions (*n* = 38) and 80 Gy in 40 fractions (*n* = 57). In the study cohort, 80 and 15 patients were elected for training/validation and testing, respectively. All patients had clinical stage T1-3N0M0 prostate cancer and were classified as high risk according to the National Comprehensive Cancer Network definitions (www.nccn.com). The patients were all manually planned and treated with eight-field IMRT; the photon energy was 15 MV, and the gantry angles were 35°, 60°, 100°, 165°, 195°, 260°, 300°, and 325°. All of the treatments were planned by using the Eclipse treatment planning system with the Anisotropic Analytical Algorithm (AAA) or Acuros XB Algorithm (AXB) to deliver 78 or 80 Gy over 39 or 40 fractions. All plans were optimized by sparing of the rectum, bladder, urethra, and femoral heads according to our department prostate radiotherapy treatment protocol, which is essentially based on QUANTEC data [8]. Clinical target volume (CTV) included the prostate and seminal vesicles, and planning target volume (PTV) was obtained by expanding CTV in three dimensions with a 0.5-cm margin. The prescribed dose was used to cover 95% of PTV-1, excluding rectum and urethra from PTV. The maximum allowable dose heterogeneity in PTV-1 was 10%. Each treatment plan was optimized to ensure the following constraints: no more than 65% of the rectal and urinary bladder wall received >35 Gy (V_35_ ≤ 65%); no more than 45% of the rectal and urinary bladder wall received >55 Gy (V_55_ ≤ 45%); no more than 25% of the rectal and urinary bladder wall received >75 Gy (V_75_ ≤ 25%); and the urethral, rectal, and bladder walls received no more than 80 Gy [34]. For PTV-2 and PTV-3, overlapping regions between the PTV and the critical organs, the constraint was set to 90% of the prescription dose. For each plan, contours of the PTV and the OARs were determined by experienced radiation oncologists, and dose distribution was optimized and confirmed by experienced medical physicists. To avoid severe late urinary toxicity following high-dose IMRT [35], a urethral catheter was used to contour and identify the urethra with planning CT image acquisition [34]. The study datasets included some cases with overlap between the PTV and a part of the large or small intestines. However, in most cases, only a few slices had overlapping regions. The training dataset included 10% (8/80) of the cohort, and the test dataset included 13% of the cohort (2/15), which had >1% and <5% overlapping regions between the PTV-1 and part of both bowels.

### Model training and validation

**Fig. 1.**
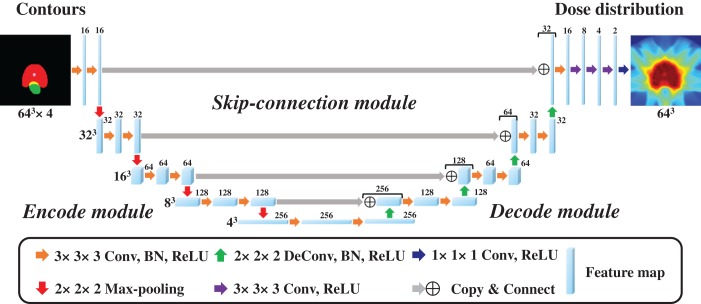
Schematic diagram of the convolutional neural network architecture used for intensity-modulated radiation therapy dose distribution prediction for input contours from planning computed tomography images.

**Fig. 2.**
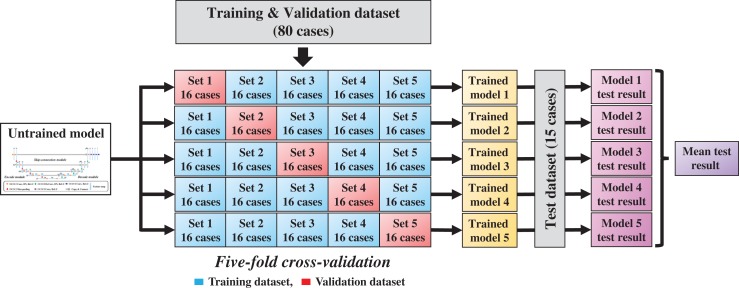
Schematic diagram of the training and evaluation processes with 5-fold cross-validation and model averaging.

In this study, a 3D CNN expanded with the similar 2D U-net [36] was employed to achieve 3D contours for 3D dose distribution mapping; the network structure is shown in Fig. 1. The architecture of the model includes three modules, encode, decode, and skip-connection modules, which are integrated into a simple network. The motivation for applying U-net was based on the following. The encode module extracts global abstraction features while reducing spatial dimension, the encode module reconstructs spatial data from features extracted by the encode module, and the skip-connection module integrates the global abstraction features and spatial features of the same size. The encode module comprises four repeated blocks of two 3 × 3 × 3 convolution layers, each followed by a batch normalization (BN) layer [37], a rectified linear unit (ReLU), and a 2 × 2 × 2 max pooling layer for the down-sampling process. The decode module also comprises four repeated blocks of two 3 × 3 × 3 convolution layers, each followed by a BN layer, a ReLU, and a 2 × 2 × 2 deconvolution layer for the up-sampling process. In the skip-connection module, we integrated the resolution information of the encoding and decoding block outputs. After the final skip-connection, the data were processed by the block comprising 3 × 3 × 3 convolution layers followed by a BN layer, a ReLU, and three 3 × 3 × 3 convolution layers followed by a ReLU, and a 1 × 1 × 1 convolution layer followed by a ReLU. All parameters of the model were globally optimized in the training stage. The input started with four channels of 64 × 64 × 64-pixel images in which we assigned the four contour binary masks to each channel, whereas the output ended with one channel of 64 × 64 × 64 dose distribution. The model was implemented with the Chainer which is part of Python’s toolbox. The contours (PTV, bladder, rectum, and urethra) and the dose distributions were obtained from the clinical radiotherapy plan. To regard the prediction accuracy of the doses for PTV and OARs that partly overlapped with PTV as important and to avoid GPU memory overflow, we selected the abovementioned four structures and reduced the volume dimensions with resolutions of 3.5 × 3.5 × 3.5 mm; we also cropped the volume data to 64 × 64 × 64. For the training process, all patient dose distributions were normalized to the mean dose of the target that was equal to 1. In the training stage, we chose the adaptive moment estimation (Adam) algorithm optimizer (α = 0.001, β_1_ = 0.9, β_2_ = 0.999, ε = 10^−8^) [38] used as training parameters, the loss function that is the difference between the predicted values and the actual values was chosen as the mean squared error between the predicted dose distribution and the clinical dose distribution, and the mini-batch size was set to four. In addition, to prevent the model from overfitting, we applied dropout layers [39] in which we tuned each rate based on the maximum rate of 0.125 to train the model successfully. To assess the abovementioned architecture and hyper-parameters, we used 5-fold cross-validation, in which 80 patients for the model training were randomly subdivided into a training set of 64 patients and a validation set of 16 patients; the validation method is shown in Fig. 2. Through this 5-fold cross-validation, the five trained models were produced. The training for the proposed model ran for 250 epochs, which took approximately 1 hour for each cross-validation step on GeForce GTX 1080 Ti 11GB GPU. The network training normally converges after about 150 epochs; we added 100 additional epochs for robustness. A curve of validation loss was used to determine that overfitting was not occurring, a well-known method to prevent overfitting [40].

### Model performance

As shown in Fig. 2, to test the model performance and reduce generalization error, we applied 15 additional patients to each trained model and averaged the test prediction results from the five trained models to acquire the mean test result. To evaluate the model performance for dose prediction, we calculated DVH parameter errors for each structure, where we compared the predicted dose distributions against the clinical dose distributions. As DVH parameters, we calculated three PTVs D_*X*_ (*X* = 2, 98), and V*xx* (*XX* = 35, 50, 65) for the rectum and bladder, which represented the percentage dose covering *X*% of the volume and percentage volume covering *XX* Gy of the dose, respectively, where all dose distributions were normalized so that D_95_ of PTV-1 was equal to the prescription dose.

### Comparison with RapidPlan

The CNN model performance was evaluated by comparing the errors against the clinical dose distributions and the dose distributions generated by RapidPlan^TM^. To train and test the RapidPlan^TM^ performance, the same 80-patient training and validation set and the 15-patient testing set were used for training and test, respectively. The RapidPlan^TM^ model was evaluated, and some possible outliers that were identified in the regression of the principal components were analyzed according to Cook’s distance (a measure of the influence of individual training set cases on regression coefficients; a score >4 indicates an influential datum that might be a geometric or dosimetric outlier) or to the Studentized residual (a score >3 could indicate a dosimetric outlier) [41]. After this process, the number of cases in the training sets was decreased from 80 to 68 patients. The model was trained on this training set from which the geometric and dosimetric outliers were removed. For each patient, plans were optimized using the generated RapidPlan^TM^ constraints provided by the model. To compare without any human intervention, a single automatic optimization process was performed with AAA as the dose calculation algorithm. Based on the prostate IMRT template of our department, we used manual optimization objectives for PTVs and a separate objective named ‘line objective’ for OARs. To evaluate the model performance of dose prediction, we calculated DVH parameter errors against the clinical dose for each structure, where all dose distributions were normalized with the abovementioned method, and we used the Wilcoxon test to determine significant differences between the CNN-clinical and RapidPlan^TM^-clinical errors using JMP statistical software (SAS Institute, Cary, N.C.).

## RESULTS

### Model performance

**Fig. 3.**
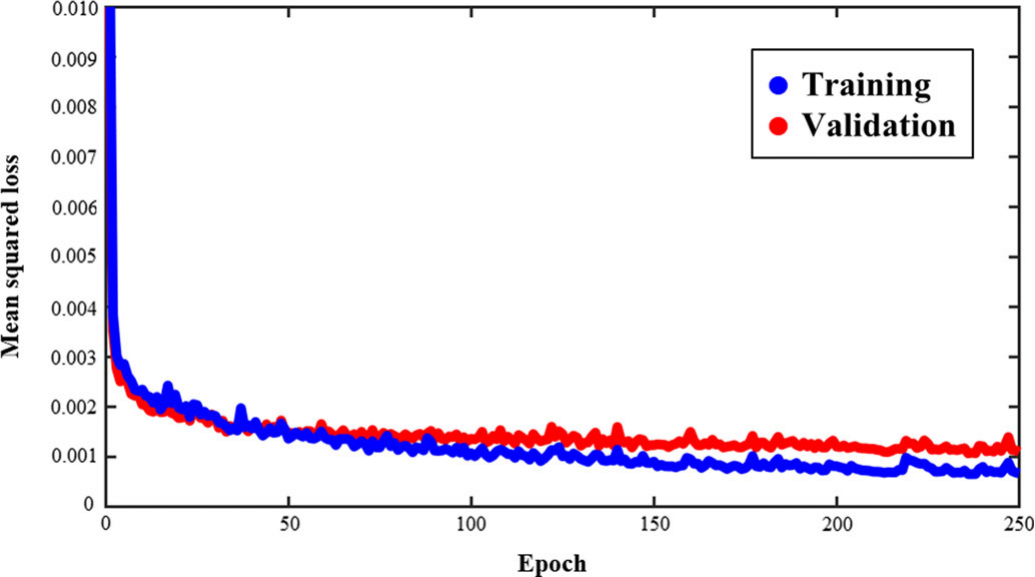
An example of a training and validation loss curve from one of the folds. Blue and red curves indicate training and validation loss, respectively.

**Fig. 4.**
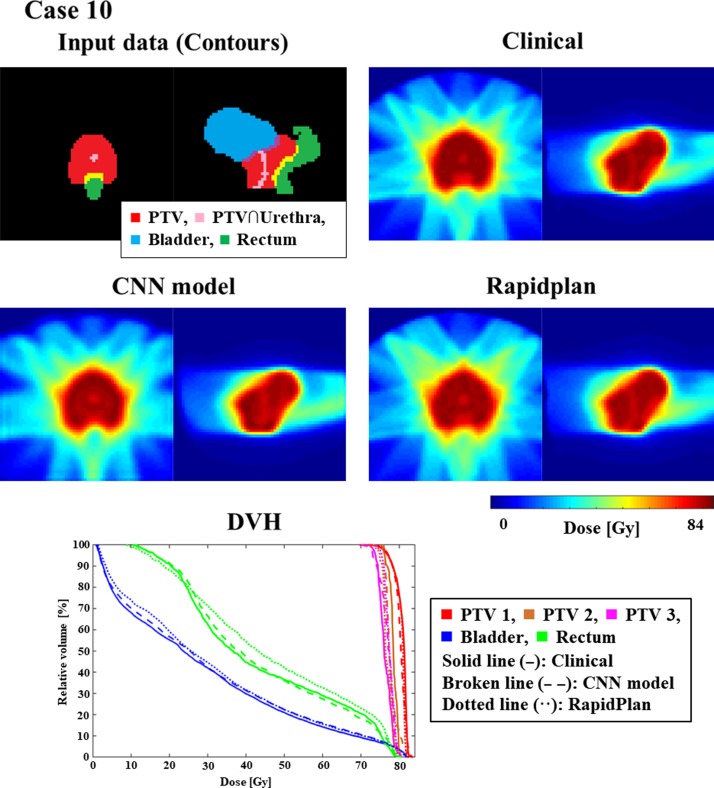
An example (Case 10) of similarly matched dose distributions predicted clinically and by CNN. ForDVHs, solid, broken, and dotted lines indicate dose distributions determined clinically and by the CNN model and RapidPlan^TM^, respectively.

**Fig. 5.**
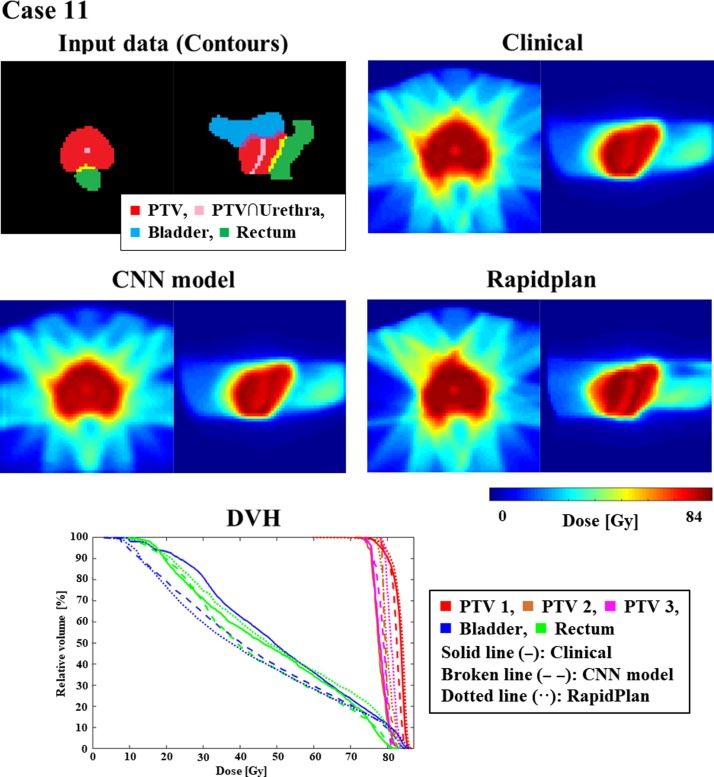
An example (Case 11) of similarly mismatched dose distributions predicted clinically and by CNN. For DVHs, solid, broken, and dotted lines indicate dose distributions determined clinically and by the CNN model and RapidPlan^TM^, respectively.

**Fig. 6.**
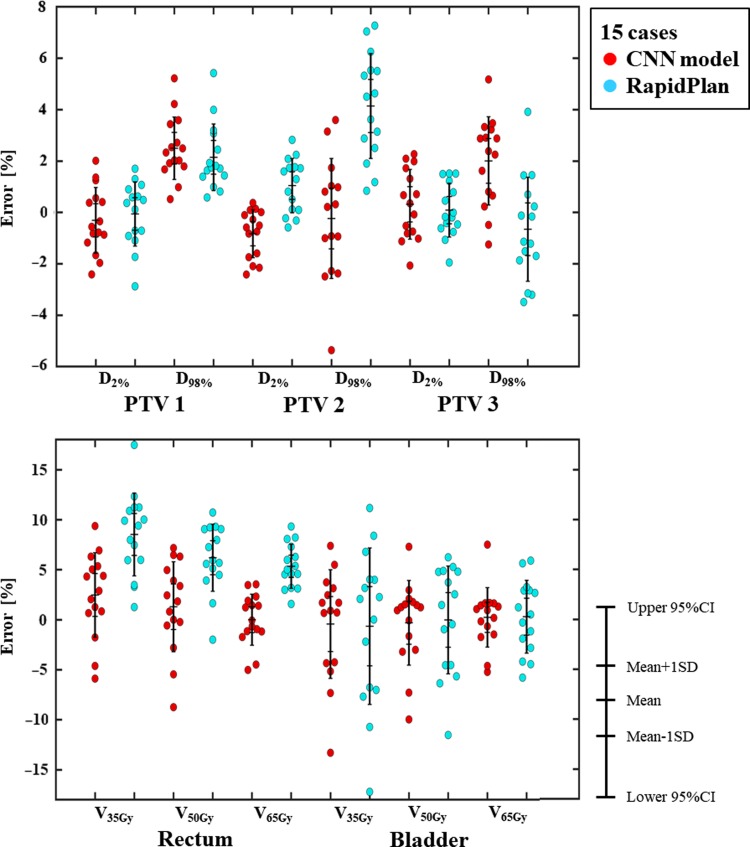
Schematic diagram of bee swarm plots for DVH parameter errors in planning target volumes (PTVs) and organs at risk (OARs). Red and blue dots indicate the errors for the predicted errors by CNN and RapidPlan^TM^, respectively.

Figure 3 shows an example of training and validation loss curve from one of the folds. The final average loss ± one standard deviation for training and validation between all the folds is 3.14 × 10^−3^ ± 8.11 × 10^−5^ and 4.90 × 10^−3^ ± 5.80 × 10^−5^, respectively. Figures 4 and 5 show the contours and DVHs of representative cases for comparisons between the CNN-predicted dose distribution and the clinical dose distribution. For example, as shown in Fig. 4, in one case (Case 10), the CNN-predicted dose distribution was similar to the clinical dose distribution, and DVH curves were closely matched by visual inspection as well. Conversely, in another case (Case 11), as shown in the example in Fig. 5, the CNN-predicted dose distribution was not similar to the clinical dose distribution. The DVH parameters absolute errors for both of the cases presented in Figs 4 and 5 are shown in Table 1. In Table 2, the mean absolute errors (MAE) with one standard deviation between the clinical and the predicted dose distributions are summarized. The MAE were within 3.00 and 5.00% for PTVs and OAR-DVH parameters, respectively. Additionally, as shown in Fig. 6, the range of signed errors was within ±6.00 and ±14.0% for PTVs and OAR-DVH parameters, respectively.

**Table 1.** Predicted absolute errors for clinical dose (%) for cases 10 (similar to the clinical dose distribution) and 11 (not similar to the clinical dose distribution)

DVH parameters	% Error with clinical dose	
	Case 10	Case 11
**PTV-1**		
**D**__**2%**__	**0.38**	**0.87**
**D**__**98%**__	**1.92**	**2.03**
**PTV-2**		
**D**__**2%**__	**0.01**	**0.74**
**D**__**98%**__	**1.00**	**0.93**
**PTV-3**		
**D**__**2%**__	**2.27**	**0.71**
**D**__**98%**__	**0.23**	**5.18**
**Rectum**		
**V**__**35Gy**__	**2.89**	**5.89**
**V**__**50Gy**__	**0.58**	**8.74**
**V**__**65Gy**__	**1.73**	**5.02**
**Bladder**		
**V**__**35Gy**__	**0.72**	**13.30**
**V**__**50Gy**__	**1.24**	**9.97**
**V**__**65Gy**__	**1.68**	**4.62**

**Table 2.** Results of the CNN-predicted and RapidPlan^TM^ errors for clinical dose (%)

DVH parameters 15 cases	% Error with clinical dose (Mean ± 1SD)		
	CNN model	RapidPlan	*P*
**PTV-1**			
**D**__**2%**__	**1.10 ± 0.64**	**1.01 ± 0.66**	**0.76**
**D**__**98%**__	**2.50 ± 1.17**	**2.15 ± 1.25**	**0.22**
**PTV-2**			
**D**__**2%**__	**0.91 ± 0.82**	**1.19 ± 0.84**	**0.37**
**D**__**98%**__	**1.81 ± 1.36**	**4.14 ± 1.97**	**<0.01**
**PTV-3**			
**D**__**2%**__	**1.17 ± 0.68**	**0.84 ± 0.57**	**0.19**
**D**__**98%**__	**2.24 ± 1.33**	**1.69 ± 1.19**	**0.33**
**Rectum**			
**V**__**35Gy**__	**4.11 ± 2.43**	**8.52 ± 4.00**	**<0.01**
**V**__**50Gy**__	**3.67 ± 2.67**	**6.46 ± 2.67**	**<0.01**
**V**__**65Gy**__	**2.04 ± 1.40**	**5.34 ± 2.13**	**<0.01**
**Bladder**			
**V**__**35Gy**__	**4.15 ± 3.24**	**6.23 ± 4.32**	**0.18**
**V**__**50Gy**__	**3.05 ± 2.75**	**4.52 ± 2.59**	**0.10**
**V**__**65Gy**__	**2.08 ± 1.99**	**3.04 ± 1.79**	**0.10**

### Comparison with RapidPlan

Table 2 shows the MAE with one standard deviation between the clinical and dose distributions generated by RapidPlan^TM^. The MAE were within 5.00 and 9.00% for the PTV and OAR parameters, respectively. Additionally, as shown in Fig. 6, the range of errors was within ±8.00 and ±18.0% for the PTV and OAR parameters, respectively. Comparison of the range of errors for CNN and RapidPlan^TM^ revealed that CNN predicted DVH parameters except for PTV-1 and PTV-3 more accurately than RapidPlan^TM^. Furthermore, our CNN model significantly predicted DVH accurately for D_98_ in PTV-2 and V_35_, V_50_, V_65_ in the rectum. Therefore, our CNN model’s prediction ability for dose distributions was superior or comparable with the dose distribution generated by RapidPlan^TM^.

## DISCUSSION

The focus of the current study was to evaluate a 3D CNN approach for prediction of IMRT dose distribution using only contours in the planning CT for prostate cancer and comparing its performance with that of RapidPlan^TM^. This is the first study to compare a CNN model with a commercial machine-learning based planning software. Our analyses revealed that our CNN model’s prediction ability for dose distributions was superior or comparable with the dose distribution generated by RapidPlan^TM^, showing its potential as an improved approach for dose distribution prediction.

As shown in Fig. 6 and summarized in Table 2, in the CNN model, the absolute mean errors were within 3% and 5 for the PTV and OAR-DVH parameters, respectively, where we used DVH parameters for each structure as evaluation indexes. Shiraishi and Moore evaluated the ability of artificial neural networks to predict 3D dose distributions for prostate cancer according to hand-crafted features such as patient-specific geometric and planning parameters including PTV, closest distance to PTV, and OARs [42]. They reported that the prediction error was less than 10% for voxels in −4 < *r*_PTV_ (distance from the PTV boundary) ≤ 30 mm. Nguyen *et al.* evaluated a CNN model’s ability to predict the dose distribution for prostate cancer based on PTV and OAR contours [30] and reported that the prediction absolute mean errors were <5.0% (max and mean dose). Although it is difficult to compare the current study results with these earlier studies directly because we used different evaluation parameters and datasets (e.g., with or without urethra dose reduction), our results were comparable with their results overall.

As shown in Fig. 6 and summarized in Table 2, our CNN model predicted DVH parameters more accurately than RapidPlan^TM^, except for PTV-1 and PTV-3. The differences might be due to the differences in the quality of features such as automatically-extracted features vs hand-crafted features. The hand-crafted features used by RapidPlan^TM^ are DVH, geometry-based expected dose, which calculates the distance between each OAR and the target surface according to the amount of dose that each target contributes to the OAR for the current field geometry, and anatomical features such as overlap volume [31]. Thus, RapidPlan^TM^ does not mutually consider relationships among the OARs and does not reflect tradeoffs between the OARs. Conversely, features automatically extracted by CNN can include not only the geometric and anatomical features such as those used by RapidPlan^TM^ but also the mutual tradeoffs between the OARs; therefore, our CNN model might have utilized important features which are still not applied in RapidPlan^TM^ (e.g., anatomical and dosimetric features) for more accurate prediction. However, while RapidPlan^TM^ can create a clinically deliverable treatment plan including several errors and limitations, such as dose calculation error, dose distribution optimization error, limitations of leaf movement, our CNN model only predicts the dose distribution. It is not clear whether the CNN-predicted dose distribution can be reproduced in a clinical setting. For accurate comparison, we will investigate whether actual dose distributions can be planned with CNN and will compare the planned dose distribution with one generated by RapidPlan^TM^.

Although our results showed the potential of CNN in predicting dose distribution, this study has several limitations. First, two different dose prescriptions (i.e., 78 Gy or 80 Gy) were used in this study. To reduce the influence on our results, dose distributions were normalized to mean dose of PTV. However, our results slightly may include the influence of dose prescription differences. Second, our datasets included some cases with overlaps between the PTV-1 and a part of the large and small intestines. While we predict that the influence of these overlaps was relatively small because the percentage of overlap between these areas was <5%, consideration of this factor could have improved the accuracy of the results to a certain extent. Third, only 95 patients were included in the current study, whereas a large dataset is typically required for deep-learning training. We consider that the sample size was not very small because the pelvic region has a relatively simple anatomical arrangement. However, a greater sample size could improve the accuracy of our analyses to a certain extent. Finally, the volume dimensions were reduced, and the volume was cropped in our dataset, leading to a lower resolution than that found in a clinical situation. However, we predict the influence of this factor on the analyses was small because the predicted dose distributions were compared with the clinical dose distributions using the same resolution. However, the lower resolution might smooth the steep dose gradient of IMRT. Thus, there remains the possibility that the predicted dose distribution might be different from the clinical plan. In future studies, we will not only predict dose distributions but also directly generate plan parameters, such as monitor-units and multi-leaf collimator movement, for plans requiring parameter optimization, such as IMRT and volumetric-modulated arc therapy. This technique for direct generation of plan parameters may improve plan consistency and planning efficiency.

## CONCLUSIONS

In this study, we compared a 3D CNN approach with RapidPlan^TM^ for prediction of IMRT dose distribution using only contours in planning CT for prostate cancer and found that our CNN model’s ability to predict dose distributions was superior or comparable with the dose distribution generated by RapidPlan^TM^, illustrating the potential utility of CNN in dose distribution prediction.
